# Characterization of a Novel *bla*_KLUC_ Variant With Reduced β-Lactam Resistance From an IncA/C Group Plasmid in a Clinical *Klebsiella pneumoniae* Isolate

**DOI:** 10.3389/fmicb.2018.01908

**Published:** 2018-08-15

**Authors:** Pingping Li, Kai Shen, Ying Zhang, Jianchao Ying, Tingyuan Zhu, Yabo Liu, Lei Xu, Chaoqing Lin, Kaibo Zhang, Peizhen Li, Junwan Lu, Kewei Li, Huiguang Yi, Qiyu Bao, Teng Xu

**Affiliations:** ^1^Institute of Biomedical Informatics, School of Laboratory Medicine and Life Science, Wenzhou Medical University, Wenzhou, China; ^2^School of Medicine, Lishui University, Lishui, China; ^3^Institute of Translational Medicine, Baotou Central Hospital, Baotou, China

**Keywords:** *Klebsiella pneumoniae*, CTX-M, KLUC enzyme, IncA/C group plasmid, IS*Ecp1*

## Abstract

Similar to other CTX-M family enzymes, KLUC is a recently identified and emerging determinant of cefotaxime resistance that has been recovered from at least three *Enterobacteriaceae* species, including *Kluyvera cryocrescens*, *Escherichia coli,* and *Enterobacter cloacae*. Whether this extended-spectrum β-lactamase (ESBL) has been disseminated among commonly isolated *Enterobacteriaceae* is worthy of further investigation. In this study, we screened 739 nosocomial *Enterobacteriaceae* isolates (240 *Klebsiella pneumoniae* and 499 *E. coli* strains) and found that one *K. pneumoniae* and four *E. coli* isolates harbored the *bla*_KLUC_ gene. Three *bla*_KLUC_ determinants isolated from *E. coli* were entirely identical to a *bla*_KLUC-3_ gene previously recovered in the same hospital. PFGE of four *bla*_KLUC_-harboring *E. coli* strains showed that prevalence of these determinants was most likely mediated by horizontal gene transfer but not clonal dissemination. However, the variant isolated from *K. pneumoniae* belonged to a novel member of the KLUC enzyme group. This newly identified enzyme (KLUC-5) has an amino acid substitution compared with previously identified KLUC-1 (G18S) and KLUC-3 (G240D). Antimicrobial susceptibility tests showed that KLUC-5 significantly reduced resistance activity to almost all the selected antimicrobials compared to previously identified KLUC-3. Site-directed mutagenesis showed that *bla*_KLUC-5_-D240G and *bla*_KLUC-5_-S18G significantly enhanced the MIC against its best substrate. Conjugation and S1-PFGE indicated that *bla*_KLUC-5_ was located on a transferable plasmid, which was further decoded by single-molecule, real-time sequencing. Comparative genome analysis showed that its backbone exhibited genetic homology to the IncA/C incompatibility group plasmids. A transposable element, IS*Ecp1*, was detected 256-bp upstream of the *bla*_KLUC-5_ gene; this location was inconsistent with the previously identified *bla*_KLUC-1_ but congruent with the variants recovered from *E. coli* in the same hospital. These data provide evidence of the increasingly emerging KLUC group of ESBLs in China.

## Introduction

CTX-M β-lactamase nomenclature is derived from their powerful cefotaxime hydrolysis activity, which is a functional indicator of these enzymes. These enzymes were initially reported in the late 1980s, and they have now become one of the most widespread ESBLs. Based on amino acid sequence similarity, the CTX-Ms are divided into at least six groups, including CTX-M-1, CTX-M-2, CTX-M-8, CTX-M-9, CTX-M-25, and KLUC (Bonnet, 2004). An increasing number of novel CTX-M variants has been identified. In addition, certain CTX-M variants have a chimeric structure, such as CTX-M-45, CTX-M-64, CTX-M-123, CTX-M-132, and CTX-M-137 (Zhao and Hu, 2013). In contrast to the many acquired β-lactamases for which the original sources remained unknown, the source of *bla*_CTX-M_ genes has been identified in certain species of the genus *Kluyvera*. In 2001, a novel chromosomally-encoded ESBL named KLUC-1 was identified from *Kluyvera cryocrescens*, and it shares 77–86% amino acid identity with other CTX-M members (Decousser et al., 2001). KLUC-2, a plasmid-mediated CTX-M family ESBL, was identified from *Enterobacter cloacae*, and it possesses a single amino acid difference compared with KLUC-1, G115R (Petrella et al., 2008). The most recently identified proteins KLUC-3 and KLUC-4 were both demonstrated to be located on plasmids from *Escherichia coli* and *E. cloacae* (Xu et al., 2012), indicating that mobile DNA elements might contribute to the transfer of the *bla*_KLUC_ gene between chromosomes and plasmids.

Acquired *bla*_CTX-M_ genes detected in clinical isolates are generally located on conjugative plasmids (Carattoli, 2009). In most cases, acquired *bla*_CTX-M_ genes are associated with either IS*Ecp1* or IS*CR1*, two different insertion sequences that are able to mobilize flanking DNA segments (Toleman et al., 2006). IS*Ecp1* is composed of an open reading frame (ORF) encoding a transposase with 420 amino acids and two imperfect inverted repeats (Bonnet, 2004). IS*Ecp1* can mobilize downstream-located *bla*_CTX-M_ genes, such as *bla*_CTX-M-3_ and *bla*_CTX-M-15_, and provide promoters for their expression (Dhanji et al., 2011; Ma et al., 2011). The IS*CR1* element is defined by an *orf513* gene encoding a putative recombinase and a recombination crossover site, and it has been identified upstream of several *bla*_CTX-M_ genes, such as *bla*_CTX-M-2_ and *bla*_CTX-M-9_, and can initiate downstream gene expression (Valverde et al., 2006). Four *bla*_KLUC_ variants have been detected in at least three genera from the family *Enterobacteriaceae*, including *K. cryocrescens*, *E. coli* and *E. cloacae*; thus whether *bla*_KLUC_ variants have been widely disseminated is worthy of further investigation. In this study, we screened for *bla*_KLUC_ variants from 240 *K. pneumoniae* and 499 *E. coli* clinical isolates and identified five bacterial strains carrying *bla*_KLUC_ variants. Among them, one was shown to encode an enzyme which is different from other KLUC group enzymes. We characterized the resistance activity and genetic environment of this new enzyme.

## Materials and Methods

### Bacterial Strains

A total of 739 consecutive, non-duplicated enterobacterial clinical isolates, including 240 *K. pneumoniae* and 499 *E. coli* strains from feces, blood, urine, and pus samples of patients, were collected in the First Affiliated Hospital of Wenzhou Medical University between 2015 and 2016. These strains were identified both by conventional methods and an auto-analysis system (BioMerieux Corporate, Lyon, France). The *bla*_KLUC-5_-harboring strain *K. pneumoniae* KP1276 was isolated from a 75-year-old male patient who was suffering upper respiratory tract and treated with cefoxitin before the bacterial strain was isolated.

### *bla*_KLUC_-Harboring Strains Identification and Conjugation Assay

PCR amplification of 739 consecutive and non-duplicated enterobacterial isolates was performed to screen for *bla*_KLUC_-positive strains. The primer sequences were described as previously (Xu et al., 2012). PCR products were sequenced on an ABI 3730XL automated sequencer (Thermo Fisher Scientific, MA, United States). The *bla*_KLUC-5_-positive strain and rifampin-resistant *E*. *coli* EC600 strain were used as the donor and recipient, respectively, to conduct conjugation assays as described elsewhere (Xu et al., 2018). The transconjugant was selected on Mueller-Hinton agar plates containing 1800 μg/ml rifampin and 512 μg/ml ampicillin. Amplification and sequencing of 16S rRNA (Forward: 5′-AGAGTTTGATCCTGGCTCAG-3′ and Reverse: 5′-GGTTACCTTGTTACGACTT-3′) and the *bla*_KLUC_ gene (Xu et al., 2012) were performed to confirm the positive transconjugants.

### PFGE, S1-PFGE and Multi-Locus Sequence Typing (MLST)

Clonal relatedness for four *bla*_KLUC_-harboring *E. coli* strains was evaluated by pulsed-field gel electrophoresis (PFGE). Bacterial DNA was extracted and then subjected to complete digestion with the restriction endonuclease *Xba*I (TaKaRa, Dalian, China). The fragmented DNA then separated in a CHEF Mapper XA system (Bio-Rad, CA, United States) at 120 V for 19 h with pulse time of 5–35 s. The DNA fingerprint patterns were analyzed according to the criteria as previously proposed (Hu et al., 2013). S1-PFGE experiments were conducted on three bacterial strains, including the wild-type KP1276, transconjugant and *E. coli* C600 (EC600). The bacterial isolates were grown on LB plates at 37°C for 16–18 h as previously reported with some modifications (Chang et al., 2013). The S1 Nuclease (TaKaRa, Dalian, China) was used to digest chromosomal DNA. *Xba*I chromosomal digestion of *Salmonella serotype Braenderup* strain H9812 was used as a molecular size marker. The digested DNA was then separated in a 1% SeaKem Gold agarose (LONZA, Rockland, ME, United States) with pulse time of 6–36 s for 18.5 h at 14°C and constant voltage of 6 V/cm. The patterns were analyzed and compared using BioNumerics software version 6.5 (Ashayeri-Panah et al., 2013). Genotyping for KP1276 was determined using 7 housekeeping genes (*gapA*, *infB*, *mdh*, *pgi*, *phoE*, *rpoB*, and *tonB*), and the MLST method was described previously (Diancourt et al., 2005). Alleles and sequence types (STs) were assigned using the MLST database^1^.

### *bla*_KLUC-5_ Gene Cloning, Site-Directed Mutagenesis and Antimicrobial Susceptibility Testing

The complete ORFs of the *bla*_KLUC_ gene from KP1276 (carrying *bla*_KLUC-5_) and D2712 (carrying *bla*_KLUC-3_) were amplified using the primers 5′-CGGGATCCATGGTTAAAAAATCATTACGCCAGT-3′ and 5′-CGGAATTCCTATAATCCCTCAGTGACGATTTTC-3′ with a pair of flanking restriction endonuclease adapters. Then, the purified PCR products were digested and ligated into the pET-28a(+) vector. The recombinant vectors were further transformed into *E. coli* BL21 using the calcium chloride method and grown on Luria-Bertani agar plates supplemented with kanamycin (50 μg/ml), IPTG (24 μg/ml), and *X*-Gal (40 μg/ml). The recombinant plasmids were verified by *BamH*I and *EcoR*I (TaKaRa, Dalian, China) digestion and sequencing. To further validate the key role of the amino acid at position 18 and 240, site-directed mutagenesis was performed to generate mutants of *bla*_KLUC-3_ and *bla*_KLUC-5_. PCR was conducted by using mutagenic primers (**Table 1**) and the recombinant clones carrying *bla*_KLUC-3_ and *bla*_KLUC-5_ as the templates which was amplified with *TransStart FastPfu* DNA Polymerase (TransGen Biotech, Beijing China). Mutagenesis was performed using a Fast Mutagenesis System (TransGen Biotech, Beijing, China). Mutations in the recombinant plasmids were confirmed by DNA sequencing. The primers used for site-directed mutagenesis were listed in **Table 1**. Bold nucleotide in the primers indicated the site-directed mutagenic base pair. A total of nine strains were subjected to minimum inhibitory concentration (MIC) detection against 17 β-lactams or their compounds. Antimicrobial susceptibility testing of selected antibiotics was performed by the agar dilution method according to the recommendations of the Clinical and Laboratory Standards Institute [CLSI] (2017) documents. The standard *E. coli* ATCC25922 was used as the quality control strain.

**Table 1:** Primers used for site-directed mutagenesis.

Mutagenesis	Direction of primer	Primer sequence (5′–3′)	Annealing temp (°C)
*bla*_KLUC-5_D240G	Forward	TAAAACCGGCAG CGGTG**G**TTACGGCACC	63
	Reverse	**C**CACCGCTGCCGGTTTT ATCGCCCACCA	
*bla*_KLUC-5_S18G	Forward	TTCCGCTG CTGGCA**G**GCAGCGTATCG	63
	Reverse	**C**TGCCAGCAGCGGAA AGACCGTCGCG	
*bla*_KLUC-3_G240D	Forward	AACCGGCA GCGGTG**A**TTACGGCACCA	63
	Reverse	**T**CACCGCTGCCGGTTTT ATCGCCCACC	

### *bla*_KLUC_-Harboring Plasmid Sequencing and Bioinformatic Analyses

The plasmid KP1276 was extracted using an alkaline lysis method as previously described (Nicoletti and Condorelli, 1993). A 20-kb library was generated using the SMRTbell Template Prep Kit (Pacific Biosciences, Menlo Park, CA, United States) according to the PacBio standard protocol and sequenced on a PacBio RS II instrument. In addition, a paired-end library with 300-bp insert sizes was constructed and sequenced from both ends using Illumina technology (Illumina, CA, United States). The PacBio long reads were initially assembled using Canu software (Koren et al., 2017). The Illumina reads were then mapped onto the assembled contigs to correct the primary assembly by using Bwa and the Genome Analysis Toolkit (McKenna et al., 2010). The potential ORFs were predicted using Glimmer software and annotated against a non-redundant protein database using the BLASTX program. The neighbor-joining phylogenetic tree of five *bla*_KLUC_ genes was constructed using MEGA6 with 1000 bootstrap replications. *bla*_CTX-M-10_ was used as an outgroup to root the tree. As mutation and selection have different effects on synonymous (*K*_s_) and non-synonymous (*K*_a_) substitution rates, the complete coding sequence of five *bla*_KLUC_ genes were pairwise alignment by using MAFFT software (*bla*_KLUC-1_ as the reference), and the *K*_a_/*K*_s_ were further measured by *K*_a__*K*_s_ calculator to understand molecular sequence evolution (Zhang et al., 2006). The three-dimensional structure of KLUC-3 and KLUC-5 was constructed via homology modeling method by using SWISS-MODEL^2^ based on the crystal structure of CTX-M-15 (Lahiri et al., 2013). The complete nucleotide sequence of the pIA/C-KLUC has been deposited to GenBank under accession number MH476540.

## Results

### *bla*_KLUC_ Gene Identification

**FIGURE 1:**
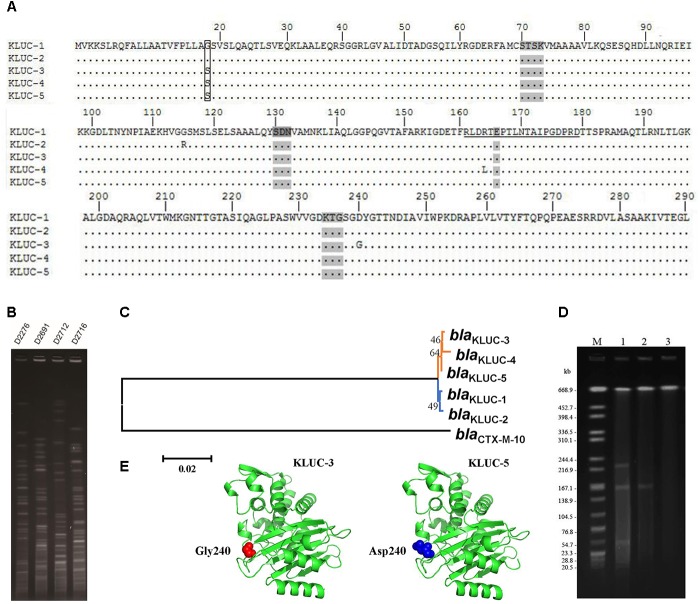
Sequence comparison, transferability and phylogenetic analyses of *bla*_KLUC-5_. **(A)** Alignment of KLUC-5 with four other KLUC subtypes. Amino acids are numbered according to the standard numbering scheme for the class A β-lactamases. Ellipses indicate identical amino acid residues. The amino acids composing the omega loop are underlined. Four structural elements characteristic of class A β-lactamases are shaded (70SXXK73, 130SDN132, 166E and 234KTG236). The substituted amino acids between KLUC-5 and KLUC-1 are boxed. The four KLUC subtypes, KLUC-1, KLUC-2, KLUC-3, and KLUC-4, were retrieved from the NCBI under accession numbers AAK08976, ABM73648, JX185316, and JX185317, respectively. **(B)** PFGE analysis of four *bla*_KLUC_-harboring *E. coli* clinical isolates. **(C)** Phylogenetic analysis of five *bla*_KLUC_ genes. Branches with the same color indicate that the genes were isolated from the same district. **(D)** S1-PFGE patterns of KP1276, transconjugant, and EC600. Lane M is the size marker strain *Salmonella serotype Braenderup* H9812 digested with *Xba*I. Lanes 1–3 are the genomes of strain KP1276, transconjugant, and EC600 digested with S1 Nuclease. **(E)** The three-dimensional structure of KLUC-3 and KLUC-5. The atoms belonging to amino acid at position 240 of KLUC-3 and KLUC-5 are indicated.

We screened the *bla*_KLUC_ gene in 240 *K. pneumoniae* and 499 *E. coli* strains via PCR. One *K. pneumoniae* (Strain No. KP1276) and four *E. coli* strains (Strain No. D2276, D2691, D2712, and D2716) were *bla*_KLUC_ positive. Sanger sequencing showed that D2276, D2691, and D2712 all harbored the *bla*_KLUC-3_ gene. In addition to *bla*_KLUC-3_-harboring strains, *E. coli* D2716 contained a *bla*_KLUC-3_-like gene that contained a stop codon at position 153. However, the *bla*_KLUC_ gene isolated from *K. pneumoniae* KP1276 belonged to a novel subtype of the *bla*_KLUC_ group enzymes. This determinant possessed 1 (S18G), 2 (S18G and G115R), 1 (D240G) and 1 (R164L) amino acid differences compared with KLUC-1, KLUC-2, KLUC-3, and KLUC-4, respectively (**Figure 1A**). Therefore, we sequentially named this new subtype as KLUC-5.

Since *bla*_KLUC-3_ has been previously identified in a clinical *E. coli* strain in the same area, the potential epidemiology of *bla*_KLUC-3_ in recently identified *E. coli* isolates would be of interest. We performed PFGE for those *E. coli* isolates which carried *bla*_KLUC-3_ (D2276, D2691, and D2712) and *bla*_KLUC-3_-like (D2716) determinants. Results showed that the genomes of four isolates exhibited distinct fingerprint among each other (**Figure 1B**), indicating that prevalence of *bla*_KLUC-3_ or its close relatives in this area is transmitted by horizontal gene transfer.

Phylogenetic analyses of five known *bla*_KLUC_ variants showed that *bla*_KLUC-1_ and *bla*_KLUC-2_, which were both isolated from Paris (France), were clustered together (**Figure 1C**), while the three other members, *bla*_KLUC-3_, *bla*_KLUC-4_, and *bla*_KLUC-5_ had closer relation and were all derived from the same district (Wenzhou, China). As *bla*_KLUC-5_ and *bla*_KLUC-3_ differed by only a single mutation (nucleotide position 725 A->G), it suggested that the presumably same origin of these two determinants.

### Transferability of the *bla*_KLUC-5_-Harboring Plasmid

Previous study showed that *bla*_KLUC-3_ was located on a conjugative plasmid. To validate the transferability of *bla*_KLUC-5_-harboring plasmid, we performed conjugative assay of wild *K. pneumoniae* KP1276. The result showed that *bla*_KLUC-5_-harboring plasmid can transfer from the donor to the recipient cells. This was further confirmed by Sanger sequencing of the PCR products from transconjugants. S1-PFGE showed that the wild-type KP1276 harbored three plasmids (**Figure 1D**). However, only the plasmid with medium size of about 180-kb from the KP1276 wild strain was transferred to the recipient (**Figure 1D**). MLST revealed that the KP1276 wild-type strain belonged to a new sequence type ST1881. These results demonstrated that the *bla*_KLUC-5_ gene was also located on a conjugative plasmid, which lay the molecular basis for rapid spread of β-lactams resistance phenotype through horizontal gene transfer.

### Resistance Activities of *bla*_KLUC_ Variants

We cloned the complete ORFs of both *bla*_KLUC-3_ and *bla*_KLUC-5_ into pET-28a vectors and transformed them separately into the *E. coli* strain BL21. The minimal inhibitory concentrations (MICs) of six strains, including the wild-type KP1276, the transconjugant of KP1276, BL21[pET28a::*bla*_KLUC-5_], BL21[pET28a::*bla*_KLUC-3_] and two blank controls, were tested against a series of 17 β-lactams or their compounds (**Table 2**). BL21[pET28a::*bla*_KLUC-3_] showed a high level of resistance to several selected antibiotics, including ampicillin and cefazolin as well as its best substrate, cefotaxime. The resistance activities of *bla*_KLUC-3_ were highly consistent with the previous observations (Xu et al., 2012). However, BL21[pET28a::*bla*_KLUC-5_] did not show resistance to several antimicrobials, such as aztreonam, ceftazidime, and ceftriaxone, but was still resistance to ampicillin and cefazolin. The resistance activity of the transconjugant of KP1276 against selected antimicrobials was universally stronger than that of the cloned *bla*_KLUC-5_, indicating that other determinants were located on the conjugative plasmid.

**Table 2:** MICs of 17 antimicrobials for 9 strains.

Antibiotics	MIC (mg/L)								
	KP1276	EC600 [pIA/C-KLUC]	EC600	BL21	BL21 [pET28a:: *bla*_KLUC-5_]	BL21 [pET28a:: *bla*_KLUC-3_ G240D]	BL21 [pET28a:: *bla*_KLUC-3_]	BL21 [pET28a:: *bla*_KLUC-5_ D240G]	BL21 [pET28a:: *bla*_KLUC-5_ S18G]
Ampicillin	>512	>512	8	1	64	64	256	256	128
Meropenem	0.06	0.06	0.06	0.06	0.06	0.06	0.06	0.06	0.06
Imipenem	0.5	0.5	0.5	0.5	0.5	0.5	0.5	0.5	0.5
Cefotaxime	32	4	0.12	<0.03	<0.03	<0.03	2	2	2
Cefotaxime + CLA^a^	64	8	0.12	<0.03	<0.03	<0.03	2	2	1
Cefotaxime + TZB^b^	2	0.06	0.06	<0.03	<0.03	<0.03	<0.03	<0.03	<0.03
Ceftazidime	8	1	0.5	0.06	0.06	0.06	0.5	0.5	0.06
Ceftazidime + CLA^a^	4	1	0.25	0.06	0.06	0.06	0.25	0.25	0.06
Ceftazidime + TZB^b^	1	0.5	0.25	0.06	0.06	0.06	0.06	0.06	0.06
Cefepime	32	4	0.12	<0.03	0.12	0.12	0.5	0.5	0.25
Cefepime + CLA^a^	32	4	0.12	<0.03	<0.03	<0.03	<0.03	<0.03	<0.03
Cefepime + TZB^b^	4	0.12	0.12	<0.03	<0.03	<0.03	<0.03	<0.03	<0.03
Piperacillin	>1024	128	4	1	16	16	32	32	16
Piperacillin + TZB^b^	64	2	2	0.5	0.5	0.5	0.5	1	0.25
Cefazolin	>512	256	4	2	32	32	64	64	64
Cefoxitin	8	8	8	2	2	2	2	2	2
Ceftriaxone	64	8	0.125	<0.03	<0.03	<0.03	0.25	0.25	0.25

^*a*^*CLA, clavulanic acid at a fixed concentration of 2 mg/L; ^*b*^TZB, tazobactam at a fixed concentration of 4 mg/L*.

Since *bla*_KLUC-5_ had S18G and D240G substitutions with *bla*_KLUC-1_ and *bla*_KLUC-3_, respectively, and the stronger resistance activities of *bla*_KLUC-1_ and *bla*_KLUC-3_ were observed when compared to *bla*_KLUC-5_ (**Table 2**), the amino acid at positions 18 and 240 might be importance for high hydrolytic activity against oxyimino-cephalosporins. To address this concern, mutants of *bla*_KLUC-3_ and *bla*_KLUC-5_ were constructed by using site-directed mutagenesis, including mutant of *bla*_KLUC-5_ at position 240 (*bla*_KLUC-5_-D240G), mutant of *bla*_KLUC-5_ at 18 (*bla*_KLUC-5_-S18G) and mutant of *bla*_KLUC-3_ at position 240 (*bla*_KLUC-3_-G240D). The MICs of *bla*_KLUC-5_-D240G evoke the resistance activity which was similar to that of *bla*_KLUC-3_, whereas opposite mutagenesis of *bla*_KLUC-3_ at position 240 (*bla*_KLUC-3_-G240D) led to the reduced activity against oxyimino-cephalosporins (**Table 2**). This indicated that amino acid at position 240 of KLUC enzyme is a key residue for hydrolytic activity against its best substrate. Homology modeling of KLUC-3 and KLUC-5 indicated that the position 240 was located on the extremity of β3 strand (**Figure 1E**) which was similar to that of CTX-M-15 (Lahiri et al., 2013). Likewise, *bla*_KLUC-5_-S18G significantly enhanced the hydrolytic activity against cefotaxime and ceftriaxone for at least 4 MIC dilution, while it slightly enhanced MICs of ampicillin, cefepime, and cefazolin compared to wild *bla*_KLUC-5_ (**Table 2**), suggesting that Gly18 is also important for KLUC enzymatic activity. Interestingly, we also observed that the resistance activities of *bla*_KLUC-5_-D240G against ampicillin, ceftazidime, cefepime and piperacillin were stronger than that of *bla*_KLUC-5_-S18G, suggesting that the hydrolytic activities of KLUC-3 against β-lactams were higher than KLUC-1.

We further performed ML estimation of *K*_a_ and *K*_s_ in pairwise sequence comparisons for five *bla*_KLUC_ variants. Results showed that all the *bla*_KLUC_ variants have ω (*K*_a_/*K*_s_) < 1, ranging from 0.09 to 0.27, indicating that purifying selection was adopted in KLUC evolution (**Table 3**). This was also reflected by three variants including KLUC-4, KLUC-5 as well as the *bla*_KLUC-3_-like gene (D2716) out of six *bla*_KLUC_ genes had no or reduced hydrolytic activity against β-lactams.

**Table 3:** Maximum likelihood estimation of *K*_a_ and *K*_s_ in pairwise sequence comparisons for five *bla*_KLUC_ variants.

Variants	*S*-Substitutions	*N*-Substitutions	*S*-Sites	*N*-Sites	*K*_a_	*K*_s_	*K*_a_/*K*_s_
*bla*_KLUC-2_	1.50797	0.500444	190.3	682.7	0.000733	0.0079242	0.093
*bla*_KLUC-3_	2.02526	2.00713	190.6	682.4	0.002941	0.0106257	0.277
*bla*_KLUC-4_	4.04959	2.00489	249.8	623.2	0.003217	0.0162113	0.198
*bla*_KLUC-5_	2.02534	1.00178	190.2	682.8	0.001467	0.0106485	0.138

### Complete Sequence of pIA/C-KLUC and Comparative Genome Analyses

**FIGURE 2:**
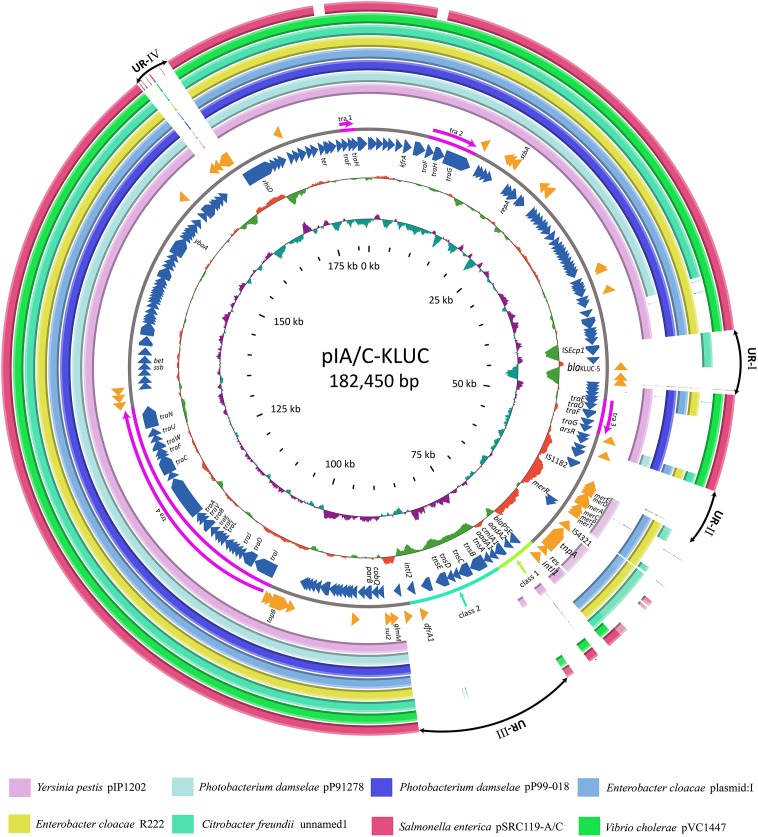
Comparative genome analyses of several IncA/C group plasmids. Structural comparison of pIA/C-KLUC with those of the most similar IncA/C group plasmids. Circles 1-8 (from outside to inside) are homologous regions of pSRC119-A/C (Accession Number KM670336), pVC1447 (KM083064), unnamed1 (CP017058), R22 (KX8697741), plasmid: 1 (LT882699), pP99-018 (AB277723), pP91278 (AB277724), and pIP1202 (CP000603) compared to pIA/C-KLUC, whereas unmatched regions are left blank. Circles 9 and 11 represent the genes encoded by the forward and reverse strands of pIA/C-KLUC. Circles 12–13 are GC content and GC skew maps of pIA/C-KLUC. The four transfer regions of the plasmid (Tra1-4) are marked in pink in circle 10 and further indicated by arrows. UR-I, II, III, and IV are four large unique regions in pIA/C-KLUC compared with the other plasmids. Classes 1 and 2 represent classes 1 and 2 integrons.

**FIGURE 3:**
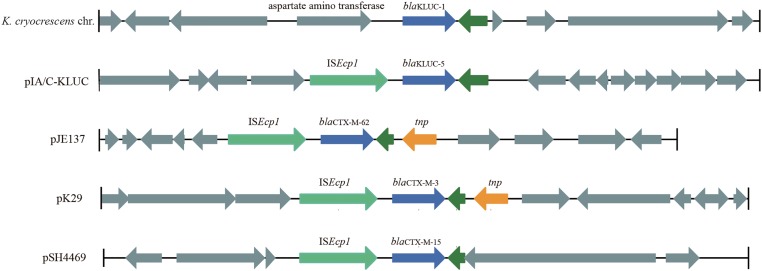
Genetic environments of several *bla*_CTX-M_ family genes. The genes are shown as arrows, with the arrowheads indicating the direction of transcription. The homologous genes are filled with the same color except for those filled with dark green. Regions are drawn to scale from accession numbers BCTM01000044 (local chromosomal fragment of *bla*_KLUC-1_), EF219134 (pJE137), EF382672 (pK29), and KJ406378 (pSH4469).

To identify the potential mobile genetic element (MGE) associated with *bla*_KLUC-5_ mobilization and the other antibiotic resistance determinants, we applied SMRT and Illumina technologies to define the complete nucleotide sequence of this conjugative plasmid. The results showed that the plasmid was 182,450 bp which had an average GC content of 51.01%, and was predicted to encode 222 ORFs. A complete nucleotide sequence search against the GenBank nucleotide database showed that the plasmid possessed the highest identity and coverage with plasmids belonging to the IncA/C incompatibility group, such as pIP1202, pP91278, pP99-018, R222, pVC1447, pSRC119-A/C and two unnamed plasmids. We designated this plasmid as pIA/C-KLUC. Comparative genome analyses showed that pIA/C-KLUC had high genomic collinearity and shared a conserved backbone with the aforementioned IncA/C plasmids (**Figure 2**). Four unique regions (UR) were detected in pIA/C-KLUC, and the genes in two of these regions (UR-II and IV) were not annotated with known functions. Interestingly, UR-III consisted of a class 2 integron which was directly subtended by a class 1 integron. The genomic architecture represented direct connection of class 1 and class 2 integrons had not been observed in recently sequenced bacterial genomes. The *bla*_KLUC-5_ gene was located 256-bp downstream of the IS*Ecp1* transposase in the UR-I of the plasmid (**Figure 2**). A comparative genome analysis of the genetic environment of several representatives of *bla*_CTX-M_ genes, including *bla*_KLUC-1_, *bla*_KLUC-5_, *bla*_CTX-M-62_, *bla*_CTX-M-3_, and *bla*_CTX-M-15_, showed that *bla*_KLUC-5_, *bla*_CTX-M-62_, *bla*_CTX-M-3_, and *bla*_CTX-M-15_ were located downstream of the IS*Ecp1* transposase, whereas *bla*_KLUC-1_ was sedentarily located on the *K. cryocrescens* chromosome (**Figure 3**). Further PCR screen of IS*Ecp1*-*bla*_KLUC_ in four *bla*_KLUC_-positive *E. coli* strains exhibited positive results, suggesting that mobilization of these *bla*_KLUC_ genes are unanimously mediated by IS*Ecp1* elements which was inconsistent with chromosomally-encoded *bla*_KLUC-1_ (Decousser et al., 2001).

## Discussion

This study identified five *bla*_KLUC_-positive strains from 739 enterobacterial isolates. Three determinants recovered from *E. coli* were demonstrated to be KLUC-3, which had been identical to previously isolated from *E. coli* D41 in the same hospital (Xu et al., 2012), indicating nosocomial dissemination of this determinant. PFGE demonstrated that prevalence of *bla*_KLUC_ in this area was transmitted by horizontal gene transfer. In addition, one *K. pneumoniae* strain was found to harbor a novel KLUC subtype, suggesting that *bla*_KLUC_ has been disseminated to the commonly isolated *Enterobacteriaceae* species in the same area. It was obvious that the clinical significance of KLUC-5 is not evident, because it possessed a reduced β-lactams resistance activity compared to previously identified variants and recently had a relative low emergence frequency. However, the epidemiology of KLUC enzyme in the present study (5/739, 0.68%) showed a significant increase in southeast of China compared to previous investigation (0.22%, 2/928) (Xu et al., 2012), and most of the KLUC variants identified in this study was KLUC-3 which was demonstrated to be a strong determinant against many β-lactam antibiotics. The amino acid sequences of five KLUC subtypes showed limited variations and only presented 1–3 substitutions among them (**Figure 1A**). Several key residues in the CTX-M family enzymes have been previously characterized. For example, Ser237 and Arg276 are specific to the CTX-M members and are used to define increased specificity for cefotaxime hydrolysis (Adamski et al., 2015). Moreover, Asn170 and Asp240 are located in the omega loop and β3 strand of the CTX-M enzymes, which are the binding regions of cefotaxime (Delmas et al., 2010). It has also been known that KLUC-1 confer strong resistance to extended β-lactams such as aztreonam and cefotaxime. We demonstrated that KLUC-3 conferred strong resistance to its best substrate, cefotaxime, whereas KLUC-4 did not show any resistance to almost all the selected antimicrobials because of a key substitution in the omega loop (Xu et al., 2012). However, the newly identified KLUC-5 which harbored only one substitution compared to KLUC-1 (G18S) and KLUC-3 (G240D), respectively, reduced the resistance activities to cefotaxime as well as most of antimicrobials. This was also validated by site-directed mutagenesis of *bla*_KLUC-3_ at position 240 (*bla*_KLUC-3_-G240D) which significantly reduced resistance activity against many antimicrobials, whereas opposite mutation of *bla*_KLUC-5_ at position 240 (*bla*_KLUC-5_-D240G) caused the increased MICs of oxyimino-cephalosporins such as cefotaxime, ceftazidime and ceftriaxone compared to wild *bla*_KLUC-5_. Indeed, D240G substitution resulted in elevated hydrolytic activity against extended β-lactams has been previously observed in other CTX-M family enzymes such as introduction of this substitution in CTX-M-14 or CTX-M-9 (Bonnet, 2004; Palzkill, 2018). Interestingly, compared to KLUC-3, KLUC-5 reduced the resistance activities against cefazolin, cefoxitin and piperacillin for no more than 1 MICs dilution, suggesting that glycine at position 240 is not required for hydrolysis of these β-lactams. However, the lack of kinetic characterization of KLUC-5 against β-lactams is a key limitation in this study.

The complete plasmid nucleotide sequence showed that *bla*_KLUC-5_ was embedded in an IncA/C group plasmid and located 256-bp downstream of IS*Ecp1*, suggesting that the movement of *bla*_KLUC-5_ was mediated by this MGE. Interestingly, an IncA/C group plasmid (‘unnamed1,’ accession: CP017058.1) isolated from *Citrobacter freundii* SL151 also harbored an IS*Ecp1* element-mediated CTX-M enzyme-encoding gene, *bla*_CTX-M-15_ (UR-I, **Figure 2**). The movement of *bla*_KLUC-5_ was similar to that of a number of CTX-M family members, such as *bla*_CTX-M-62_, *bla*_CTX-M-3_, and *bla*_CTX-M-15_, but was dissimilar to *bla*_CTX-M-2_ and *bla*_CTX-M-9_, whose mobilization is mediated by IS*CR1* (Eckert et al., 2006). In addition to *bla*_KLUC-5_, the plasmid pIA/C-KLUC also contained other antibiotic resistance genes that are included in tail-to-tail directly connected class 1 and class 2 integrons. Four antibiotic resistance gene cassettes, which were sequentially arranged as *bla*_PSE-1_, *aadA2*, *cmlA1,* and *aadA1*, were embedded in the class 1 integron, which represented a novel cassettes arrangement. This class 1 integron harbored a complete 5′-CS but lacked the entire 3′-CS, which was presumably truncated by a class 2 integron. The acquisition of the class 2 integron into pIA/C-KLUC was most probably involved the Tn*7* transposon encoded by *tnsABCDE* through horizontal transfer, which could be reflected by the biased GC skew in class 2 integrons compared with the flanking regions (UR-III, **Figure 2**), and the class 2 integrons have been illustrated to be most frequently associated with Tn*7* derivatives (Peters and Craig, 2001). In addition, a Tn*3* family transposon was also identified in pIA/C-KLUC. This transposon was possibly truncated by a gene cluster corresponding to mercury resistance flanked by a pair of insertion elements, IS*4321* and IS*1182*, and the typical inverted repeats belonging to the Tn*3* transposon were not identified in this region.

## Conclusion

Increased emergency of KLUC group enzyme-encoding genes from *Enterobacteriaceae* was observed in the southeast of China. Of them, a new subtype, KLUC-5, was recovered from a clinical *K. pneumoniae* strain and demonstrated to be located on an IncA/C group plasmid. The mobilization of *bla*_KLUC-5_ was associated with the IS*Ecp1* element, which may be different from that of *bla*_KLUC-1_ but similar to that of *bla*_KLUC-3_ from the same district as *bla*_KLUC-5_. This new enzyme showed significantly reduced resistance to extended β-lactams presumably ascribed to the two combined amino acids substitution compared to KLUC-1 and KLUC-3.

## Data Availability

Data and materials have been provided in the main manuscript. Where necessary additional information of the study can be made available from the corresponding author on request.

## Author Contributions

TX and QB designed and supervised the study. PPL, KS, YZ, JY, TZ, YL, LX, and CL performed the experiments. TX, JY, and HY analyzed the data. KZ, JL, PZL, and KL contributed the reagents. PPL drafted the manuscript. TX and QB revised the manuscript. All authors read and approved the final manuscript.

## Conflict of Interest Statement

The authors declare that the research was conducted in the absence of any commercial or financial relationships that could be construed as a potential conflict of interest.

## Footnotes

1 https://pubmlst.org/mlst/

2 https://www.swissmodel.expasy.org/
